# Retraction Note: MicroRNA-375 released from extracellular vesicles of bone marrow mesenchymal stem cells exerts anti-oncogenic effects against cervical cancer

**DOI:** 10.1186/s13287-024-03666-8

**Published:** 2024-02-27

**Authors:** Feng Ding, Jinhua Liu, Xiaofei Zhang

**Affiliations:** 1 Department of Education and Teaching, Linyi People’s Hospital, Linyi, 276000 People’s Republic of China; 2 Department of Gynecology and Obstetrics, Linyi People’s Hospital, Linyi, 276000 People’s Republic of China; 3 The 3rd Department of Gynecology, Linyi People’s Hospital, No. 27, East Section of Jiefang Road, Lanshan District, Linyi, 276000 Shandong Province People’s Republic of China

Stem Cell Research & Therapy (2020) 11:455

10.1186/s13287-020-01908-z

Retraction note

The Editors-in-Chief have retracted this article. After publication, concerns were raised regarding similar features between this article and a number of other articles on non-coding RNAs from different research groups published within a similar time frame [1–4]. Specifically, the ruler in Fig. 7A appears highly similar to that in the tumour images presented in [1–4]. The shapes and backgrounds on the western blots presented in all figures in this article also appear highly similar to those in the aforementioned articles. Additionally, Fig. 3D NC-inhibitor and miR-375 inhibitor images appear highly similar.

The Editors-in-Chief therefore no longer have confidence in the presented data.

None of the authors have responded to any correspondence from the editor or publisher about this retraction.
